# Multimodal imaging of papillary fibroelastoma

**DOI:** 10.1093/ehjimp/qyag133

**Published:** 2026-10-21

**Authors:** Lady Diana Quintana Reusche, Kevin Velarde-Acosta, Luis Falcón-Quispe, Angela Cachicatari-Beltran, Kelly Cupe-Chacalcaje, Dante Urdanivia-Ruiz, Eliana Rafael-Horna, Robert Lozano Purizaca, Roberto Baltodano-Arellano

**Affiliations:** Resident of Cardiology Service, Guillermo Almenara Irigoyen Hospital—EsSalud, Lima, Peru; Resident of Cardiology Service, Guillermo Almenara Irigoyen Hospital—EsSalud, Lima, Peru; Cardiac Imaging Area of Cardiology Service, Guillermo Almenara Irigoyen Hospital—EsSalud, Lima, Peru; Cardiac Imaging Area of Cardiology Service, Guillermo Almenara Irigoyen Hospital—EsSalud, Lima, Peru; Cardiac Imaging Area of Cardiology Service, Guillermo Almenara Irigoyen Hospital—EsSalud, Lima, Peru; Cardiac Imaging Area of Cardiology Service, Guillermo Almenara Irigoyen Hospital—EsSalud, Lima, Peru; Cardiac Imaging Area of Cardiology Service, Guillermo Almenara Irigoyen Hospital—EsSalud, Lima, Peru; Thoracic and Cardiovascular Surgery Department, José Cayetano Heredia Hospital—EsSalud, Piura, Perú; Cardiac Imaging Area of Cardiology Service, Guillermo Almenara Irigoyen Hospital—EsSalud, Lima, Peru; School of Medicine, Universidad Nacional Mayor de San Marcos, Lima, Peru

**Keywords:** papillary fibroelastoma, tumor, multimodal imaging

## Abstract

Papillary fibroelastoma is a rare primary cardiac tumor with a benign histology. However, it can cause serious complications, primarily thromboembolism to the brain. Multimodal imaging is important for tumor detection, tissue characterization, assessing complications, planning surgery, and postoperative follow-up. In summary, multimodal imaging plays an essential role in managing papillary fibroelastoma and is crucial in determining interventional decisions.

## Graphical Abstract

**Graphical Abstract:**
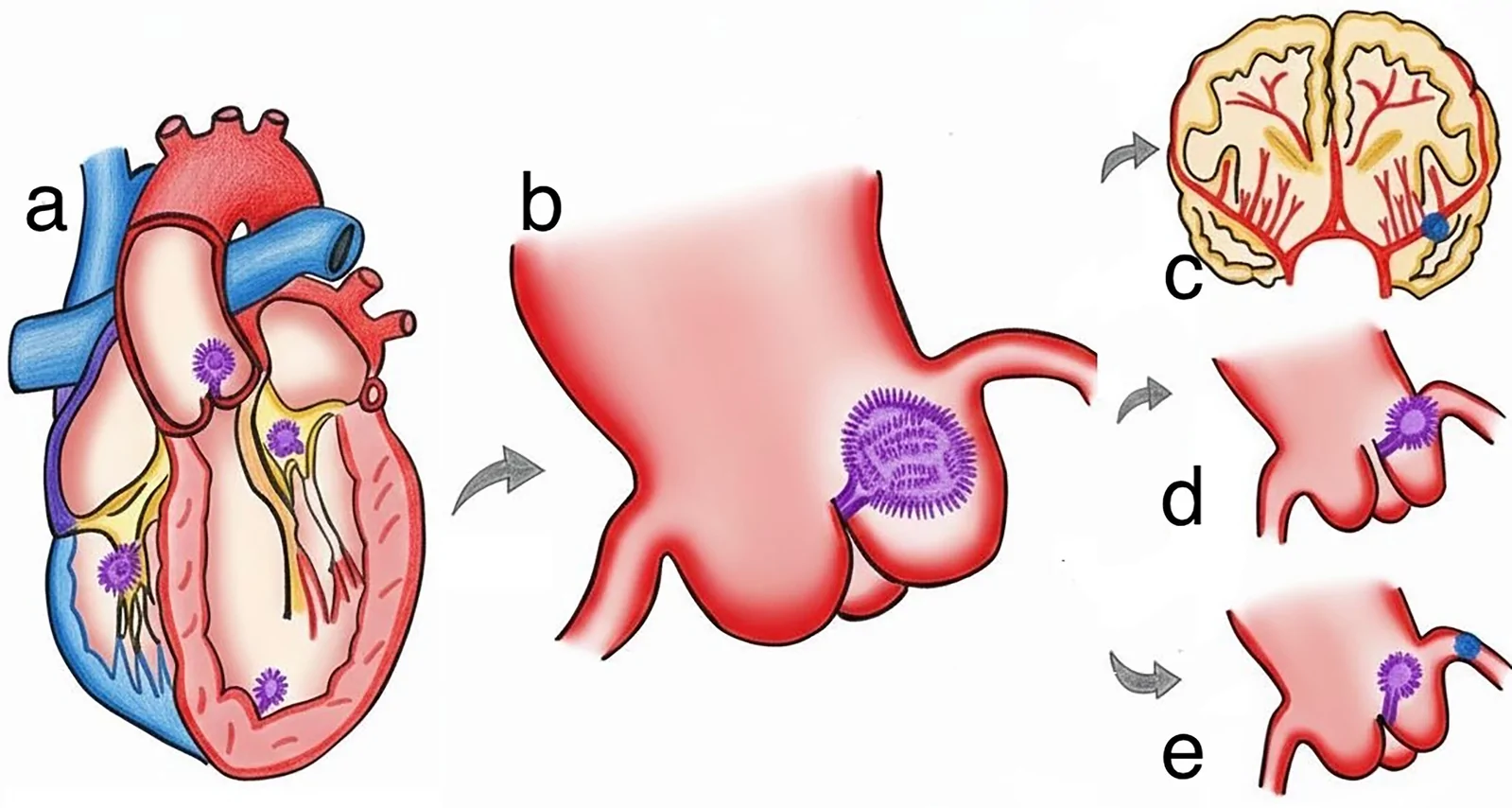
Locations of papillary fibroelastoma, the most frequent being the aortic valve (a). Small, ovoid, pedunculated tumour, located on the arterial aspect of the aortic valve (b). Embolization of part of the tumour or thrombi could cause stroke (c) and myocardial infarction (d). A large, hypermobile tumour can dynamically obstruct the nearest coronary artery during systole (e).

## Introduction

Papillary fibroelastoma (PFE) is a rare primary cardiac tumour that accounts for approximately 0.02% of all autopsy cases and about 8% of all diagnosed primary cardiac tumours.^1–3^ Though rare, it is the second most common primary cardiac tumour after myxoma. It is recognized for its potential to cause thromboembolic complications despite its benign histological nature.^3^ In most cases, the PFE is discovered incidentally. However, strokes or transient ischaemic attacks occur in 7–25% of cases, and angina is reported in up to 10% (Central Illustration).^3–5^

Although echocardiography has traditionally been the cornerstone of diagnosis, multimodal imaging, including cardiac computed tomography and cardiac magnetic resonance imaging, provides a more comprehensive understanding of the tumour’s anatomy, histology, and pathophysiology (*Table 1*). In other words, multimodal imaging is essential for diagnosing PFE, evaluating complications, making decisions, and planning treatment (*Figures 1–4*).

**Figure 1:**
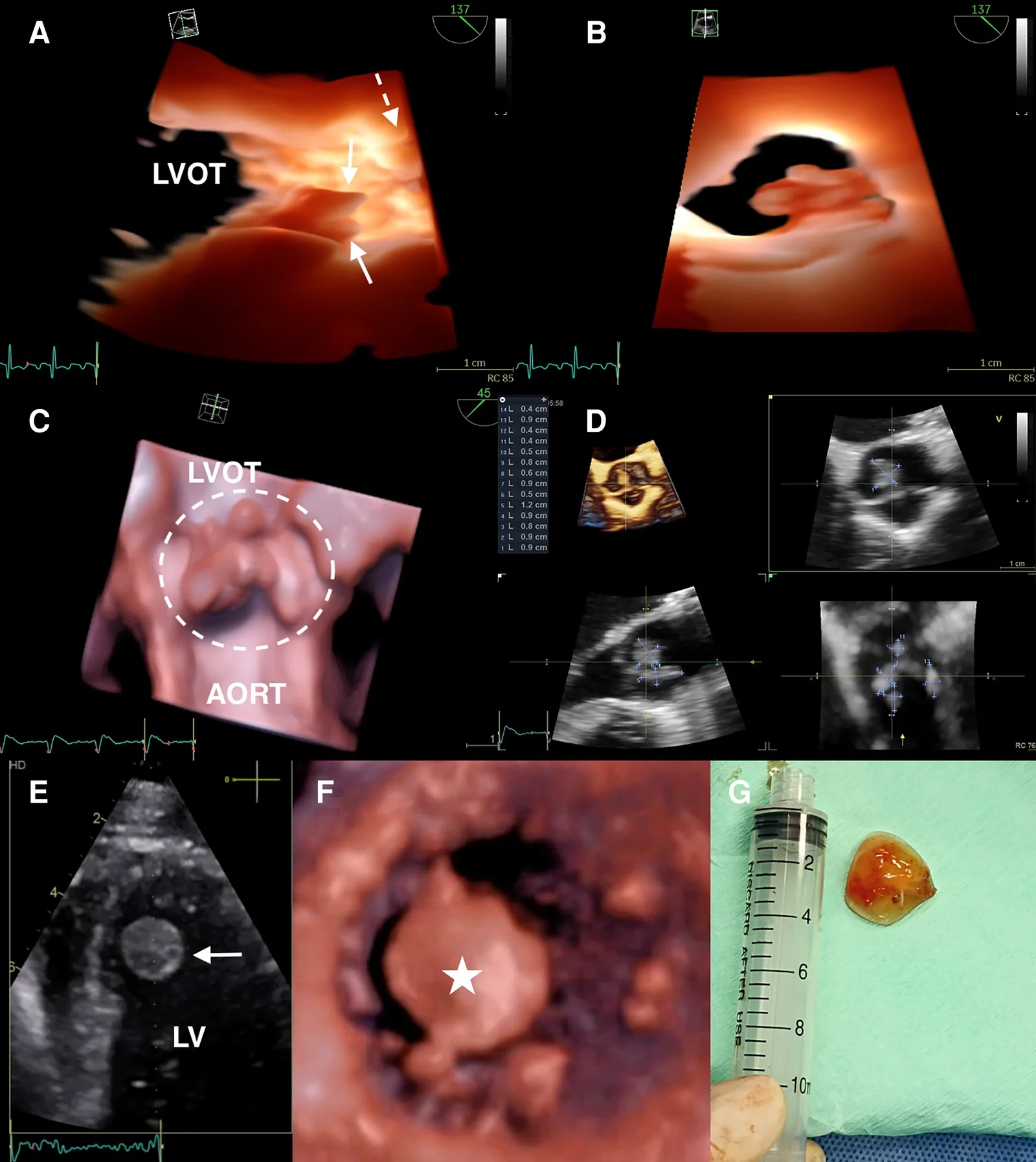
Echocardiography. (*A*) TEE, Volume rendering, transillumination. Long axis, zoom in on the LVOT. Bilobed tumour implanted in the left ventricular outflow tract (white arrows) 10 mm from the aortic valve level (dashed arrow). (*B*) Continuation of (A) Volume rendering, transillumination. Zoom in on the left ventricular outflow tract in short axis, viewed from the distal outflow tract. Multi-lobed tumour occupying approximately one-third of the corresponding transverse plane. (*C*) TEE, Volume rendering. Zoom in on the aortic root, superior view. Multiple multi-lobed tumours on the aortic cusps (dashed circle). (*D*) Continuation of C. TEE, Multi-planar reconstruction of case C. Aortic root in short axis. Lobes located on the three aortic cusps are quantified and measured. (*E*) 2D TTE. Four-chamber view. Single ovoid tumour at the apex of the left ventricle (white arrow). (*F*) Continuation of (E) TTE, Volume rendering. Four-chamber view. Pedunculated tumour at the ventricular apex (asterisk). (*G*) Macroscopic view of tumour removed from the same patient. Ovoid mass with a gelatinous surface and a solid central body, 13 mm in its largest diameter. LVOT, left ventricular outflow tract; AORT, aorta; LV, left ventricle.

**Figure 2:**
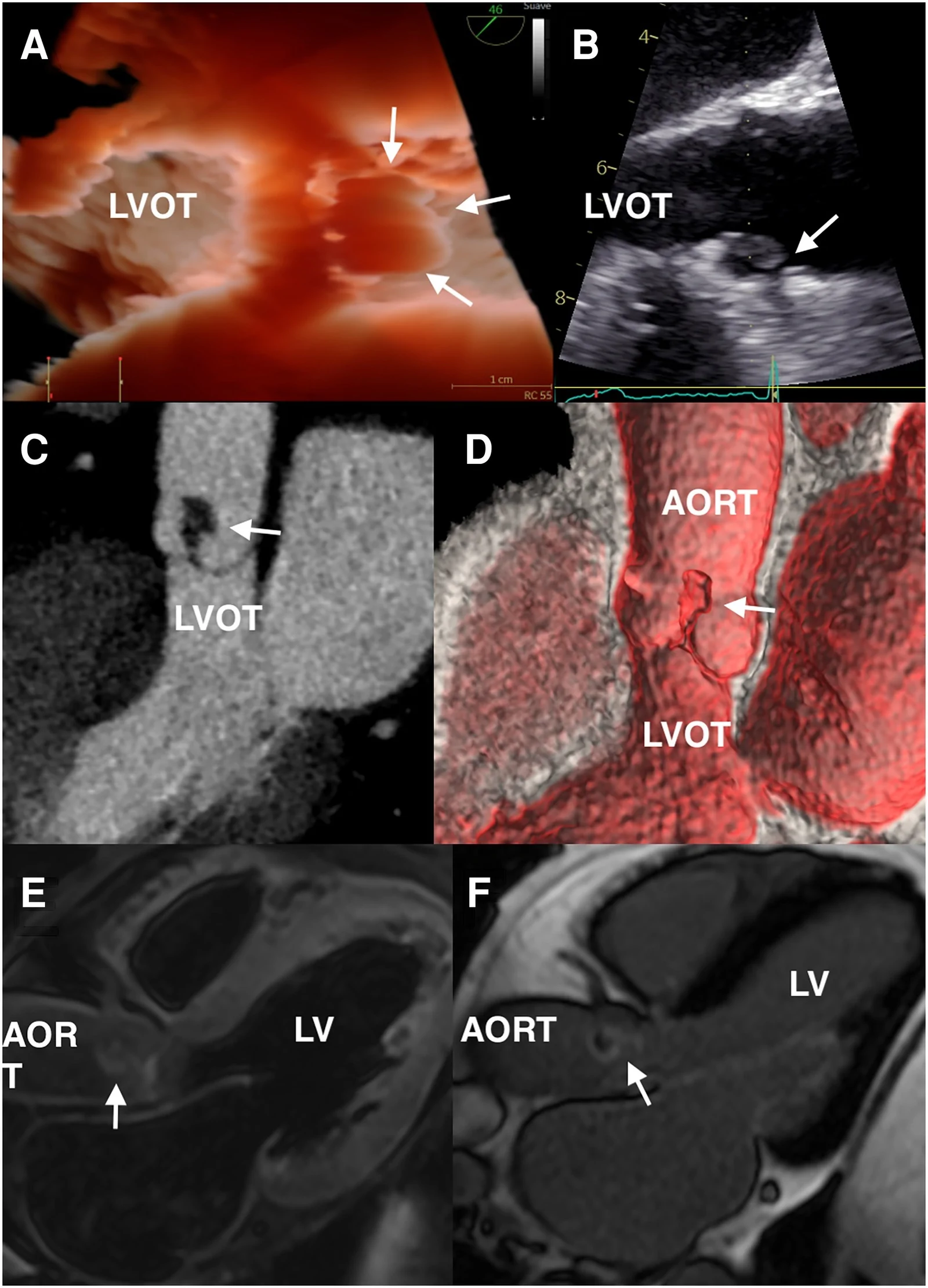
Multimodal image. (*A*) TEE, Volume rendering, transillumination. Long axis, zoom on the aortic root. Three tumours arising from the right coronary cusp and non-coronary cusp (white arrows). (*B*) 2D TEE. Long axis, zoom on the aortic root. The distal portion of the tumour obstructs the ostium of the right coronary artery during systole (white arrow). (*C*) Three-chamber cardiac CT. A hypodense small mass with a thin stalk and multiple frond-like projections arising from the aortic side of the non-coronary cusp (white arrow). These findings are characteristic of papillary fibroelastoma. (*D*) 3D rendering CT. Demonstrates a pedunculated, rounded mass with greater precision of its anatomical relationships in the aortic sinuses (white arrow). (*E*) CMR SSFP. A hyperintense mass on T2-weighted sequences (white arrow). *(F*) CMR, LGE. Gadolinium enhancement of marginal ring pattern (white arrow). LVOT, left ventricular outflow tract; AORT, aorta; LV, left ventricle.

**Figura 3:**
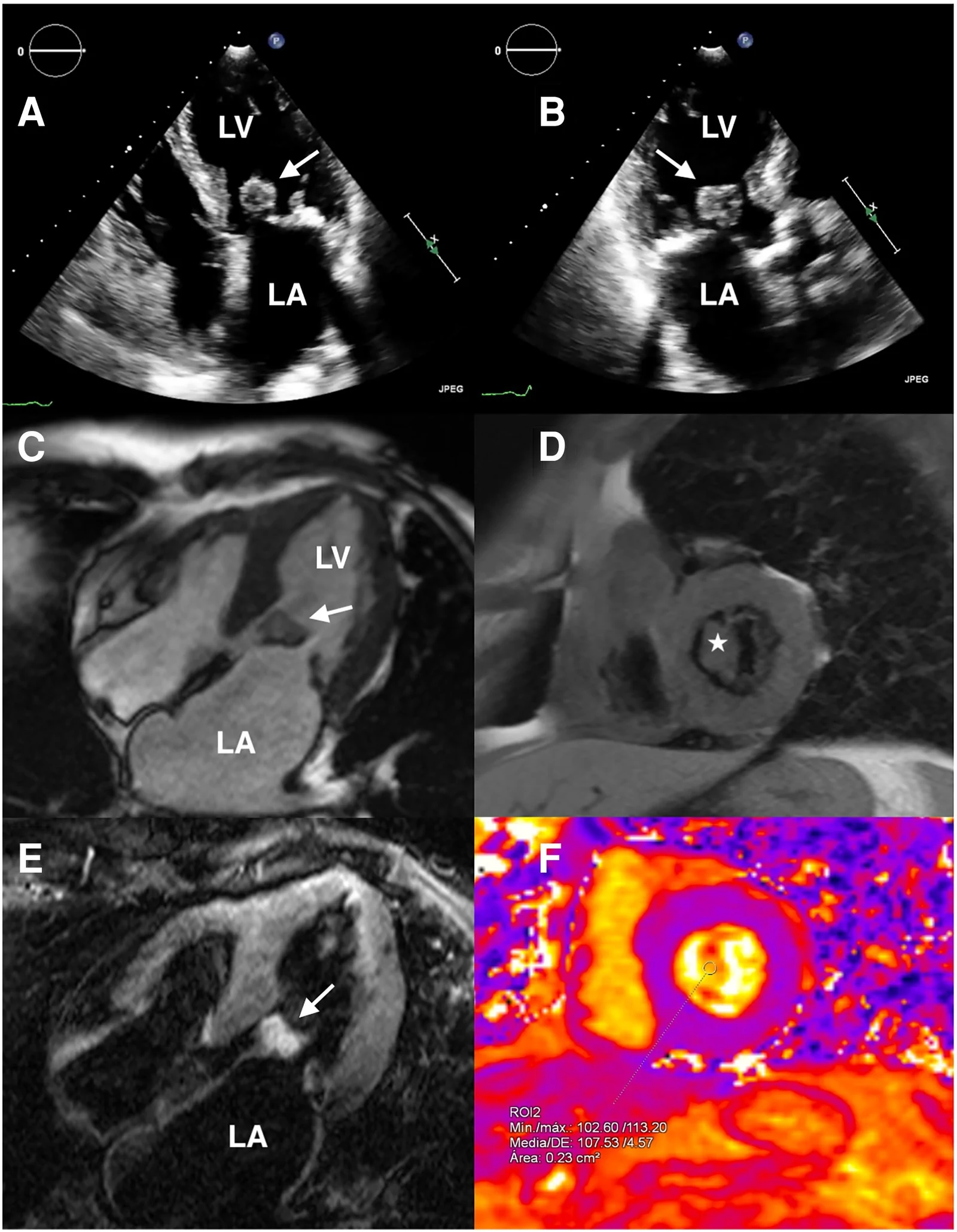
Imagen multimodal. (*A*) 2D TTE. Four cameras. Ovoid mass with irregular edges on the ventricular side of the anterior mitral leaflet (white arrow). (*B*) 2D TTE. Three cameras. Same mass with a more multi-lobulated appearance (white arrow). (*C*) CMR, Four-chamber SSFP. A small, rounded mass arising from the ventricular side of the mid-anterior mitral leaflet (white arrow). (*D*) CMR, Short-axis T1w. A small, isointense ovoid lesion arising from the A2 scallop (asterisk). (*E*) CMR, Four-chamber T2w demonstrated a small, high signal intensity mass (white arrow). (*F*) CMR, T2 mapping sequence shows a mass with high relaxation time (107 ms). LV, left ventricle; LA, left atrial.

**Figure 4:**
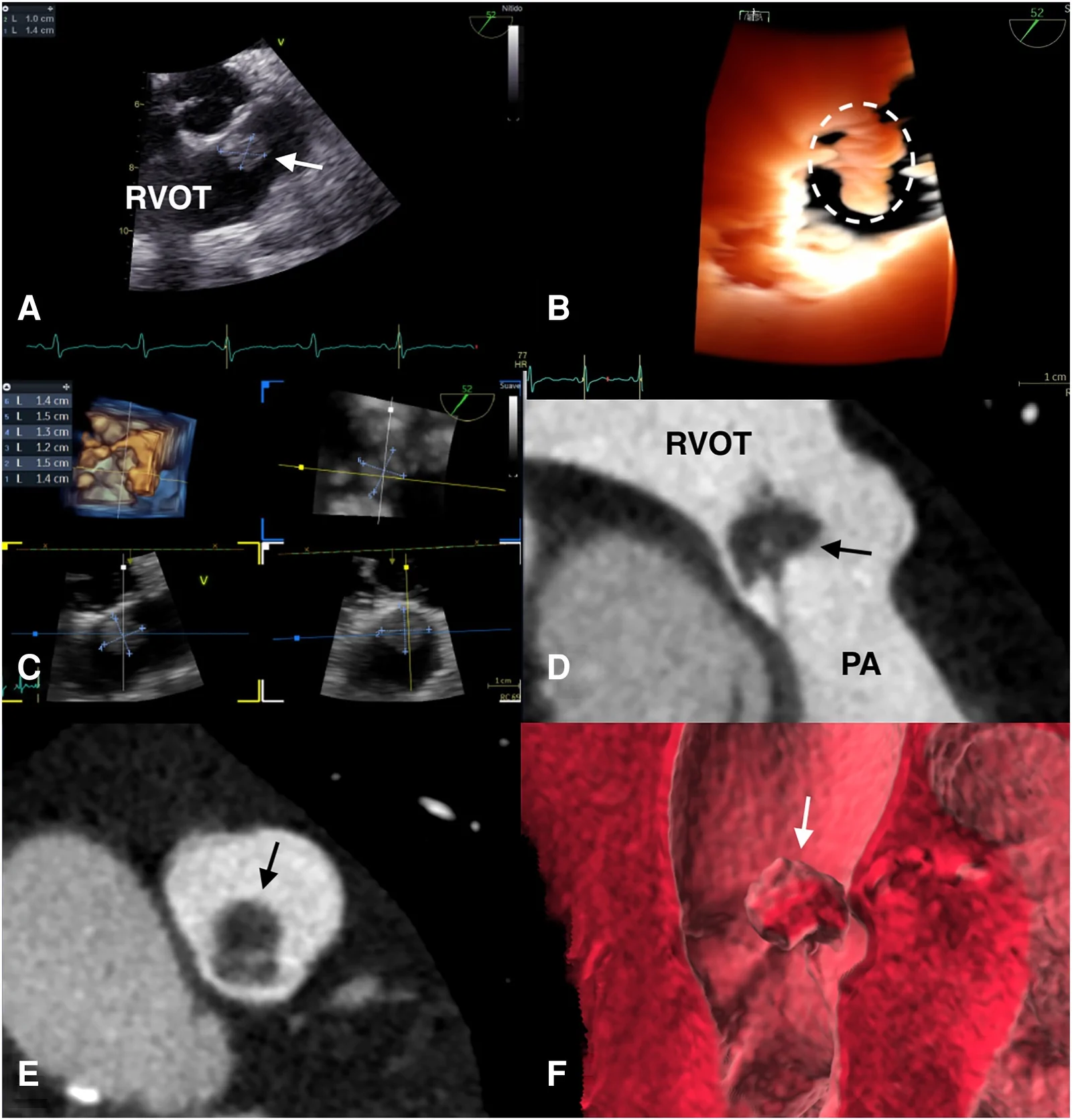
Multimodal image. (*A*) 2D TEE. Short axis, pulmonary valve focus. Ovoid mass of 14 mm arising from the pulmonary valve cusp (white arrow). (*B*) TEE, Volume rendering, transillumination. Short axis, pulmonary valve focus. Pedunculated ovoid mass with a multi-lobed appearance (dashed circle). (*C*) TEE, Multi-planar reconstruction. Short axis, pulmonary valve focus. More precise dimensions (maximum diameter 15 mm) and implantation location (left semilunar cusp). (*D*) Sagittal reformat CT. Mobile rounded mass with a multi-lobed surface arising from the pulmonary valve (black arrow). It is connected via a thin stalk to the pulmonary cusp. (*E*) Double oblique cardiac CT. A hypotenuated mass dependent on the left posterior sinus (black arrow). (*F*) 3D volume rendered CT. A photorealistic image of papillary fibroelastoma, a rounded mass prolapsed into the right ventricular outflow tract (white arrow). RVOT, right ventricular outflow tract; PA, pulmonary artery.

**Table 1:** Typical finding, advantages and disadvantages of imaging technique

	Typical findings	Advantages	Disadvantages
TTE	An ovoid mass smaller than 20 mm, homogeneous texture, pedunculated, and freely mobile	Lower cost. Greater accessibility. No radiation required. High spatial and temporal resolution in real time for structures.	Visualization and evaluation depend on the probe and the acoustic window. Reduced capacity to assess structural and functional impairment of the pulmonary valve. Operator-dependent.
TEE	Can detect PFE smaller than 5 mm and better define its pedicle, dotted perimeter and vibratory movement.	Higher spatial and temporal resolution than TTE for evaluating tumours, surrounding structures, and potential hemodynamic compromise. Suitable for use during surgical procedures.	Visualization and evaluation depend on the probe and acoustic window. Reduced capacity to assess structural and functional impairment of the pulmonary valve. Operator-dependent. Sedation is required in some cases.
3D TEE	Realistic image of the PFE and its anatomical relationship.	Provides a highly realistic depiction of the structures being evaluated. Multi-planar reconstruction accurately determines the dimensions and location of the tumour.	The presence of acoustic artefacts and a suboptimal acoustic window can limit the 3D visualization of structures. Acquiring images with a higher spatial and temporal resolution requires a constant heart rate and breath-holding.
CT	Small, hypodense masses with tentacle-like edges due to the presence of a pedicle.	High spatial resolution. Using multi-planar imaging, it provides visualization of tumour movement and involvement of adjacent structures. Particularly useful in the study of coronary arteries. Allows for the visualization of calcified components.	Lower temporal resolution. Ionizing radiation. Limited use of iodinated contrast in patients with renal insufficiency. Requires low heart rate.
CMR	Several sequences show the anatomy of the tumour with exceptional spatial resolution. From a histological standpoint, point toward a diagnosis of PFE	Allow tissue characterization of tumours. Use of contrast agents, such as gadolinium, allows for the assessment of vascularization and thus helps determine whether a lesion is benign or malignant. Allow the evaluation of the dynamic relationship and impact on valvular structures.	Higher cost. Limitation of gadolinium use in patients with renal insufficiency. Long time to acquire and requires breath-holding.

CMR, cardiac magnetic resonance; CT, computed tomography; PFE, papillary fibroelastoma; TEE, transoesophageal echocardiography; TTE, transthoracic echocardiography.

In this article, we will review the nature and consequences of PFE. Our main focus will be on the contribution and limitations of imaging techniques in evaluating this tumour, and how integrating these modalities improves diagnostic accuracy and guides clinical decisions. Finally, we will present cases in which multimodal imaging illustrates PFE.

## Aetiopathogenesis, histology, and pathophysiology

The aetiology is unclear, but two hypotheses have been proposed. One hypothesis is that the tumour originates from valvular endothelial damage that occurs around the sixth and eighth decades of life. This is consistent with the detection of the tumour in patients undergoing cardiac surgery.^6^ On the other hand, the genuine neoplastic theory is based on mutations in the KRAS oncogene, which occur in up to 80% of cases.^7^

Macroscopically, the PFE has an ovoid, gelatinous appearance with a smooth surface. However, when placed in saline solution, it takes on a whitish, arborized appearance with short branches and a central stem, resembling a sea anemone.^8^ Microscopically, the tumour is poorly vascularized and composed of a central core of collagen, elastin, and reticulin. The middle layer is formed by mucopolysaccharide, and multiple layers of endocardial cells comprise the outer wall.^9^

The papillary structure and high mobility of these tumours allow fragments or clots to break off. This could lead to embolic events and the potential occlusion of distal vessels, resulting in ischaemia or infarction of vital organs such as the brain and the myocardium.^10^ Ischaemic strokes are the most common complication, accounting for up to 25% of cases in one series.^3^ The second most common complication of PFE is coronary obstruction, whether due to dynamic obstruction of the coronary ostium by the tumour or due to thrombi from the tumour that embolize into the coronary arteries, resulting in angina, myocardial infarction, ischaemic ventricular tachycardia, and even sudden death.^3–5,11^ Likewise, there have been anecdotal reports of PFE leading to aortic valve disease, either aortic valve insufficiency or stenosis.^12^

## Echocardiography: detection and morphological characterization

Transthoracic ultrasound is the initial diagnostic imaging technique. Key morphological characteristics include: an ovoid mass smaller than 20 mm, homogeneous texture, pedunculated, and freely mobile.^9,13^ When the pedicle and the homogeneous echogenicity of the collagen core is visualized, it is possible to differentiate it from other tumours.^3^ The accuracy of the test for masses ≥0.2 cm is 89% sensitivity and 88% specificity, with an overall accuracy of 88.4%.^13^ False negatives may be due to lesions smaller than 2 mm, masking by associated lesions, or an evaluation conducted with a low level of suspicion.

PFE primarily occur in the aortic valve (63%), particularly on the arterial side of the valve and in the non-coronary leaflet, followed by the mitral valve and, less frequently, the tricuspid and pulmonic valves.^2,3^ These tumours have also been found in non-valvular structures, including the atrial and ventricular walls, Marshall’s ligament, the sinus of Valsalva, aortic wall, and pulmonary artery.^14^

Unlike transthoracic echocardiography, transoesophageal echocardiography (TEE) offers better spatial and temporal resolution and allows for the detection of masses smaller than 5 mm. TEE also shows a better-defined stalk and a bright, dotted perimeter, as well as the typical vibratory movement of the border.^9,13,15^ Furthermore, TEE generally demonstrates that PFE does not lead to valvular or adjacent structure damage, which helps distinguish it from other cardiac masses such as endocarditis or malignant tumours.^15^

Three-dimensional TEE (3D TEE) provides highly realistic images of PFE and its related anatomy, especially when the transillumination tool is included, as it enhances the depth effect. In addition, multi-planar reconstruction accurately determines the size, location, morphology, and mobility of the tumour.^16^

The anatomical and functional information provided by echocardiography enables differentiation of this tumour from other pathologies. The presence of inflammatory and aggressive changes points towards a diagnosis of vegetations, while a stasis context suggests thrombosis.^3^ Myxomas are typically larger, have a heterogeneous morphology and echogenicity, and are usually located in the left atrium, attached to the fossa ovalis. Finally, Lambl excrescences frequently appear along the cusp coaptation line, are usually multiple, and consist of a single outer layer of endothelial cells, unlike PFE, which has multiple layers of these cells.^8^

## Magnetic resonance imaging: a histological approach

The main advantage of this technique over other imaging modalities is its ability to provide histological information, making it an essential tool for the study of cardiac masses. Furthermore, its high spatial resolution allows for optimal anatomical evaluation. Due to the various sequences available, it is useful for diagnosis, assessing the functional impact of the lesion, pre-surgical planning, and post-treatment follow-up.^17,18^

The cine sequence (SSFP, or steady-state free precession) enables evaluation of a mass’s mobility and attachment point, as well as its dynamic relationship with and impact on valvular structures. PFE typically appear as hypointense, pedunculated, mobile, and well-circumscribed masses, usually attached to the valvular endocardium by a short, thin pedicle. It is worth noting that the size of the fibroelastoma sometimes limits its detection using this imaging modality.^19^

On the other hand, black-blood sequences allow for evaluation of tissue composition. T1- and T2-weighted relaxation properties, as well as differences in hydrogen proton density among different tissues, allow for the differentiation between normal structures, benign lesions, and malignant lesions.^18^ T1- and T2-weighted images show intermediate and hyperintense signals, respectively, reflecting the primarily fibroelastic composition of this tumour.^17,20,21^ Likewise, T1 mapping is characterized by increased relaxation times.^16^ The addition of fat suppression to these sequences helps differentiate PFE from a mass with a fatty component.^22^

Finally, first-pass perfusion and late gadolinium enhancement sequences evaluate vascularization and delayed gadolinium washout in tissues, which is useful in distinguishing between benign and malignant masses.^17^ Since PFE are poorly vascularized tumours, there is usually no first-pass or late enhancement. However, there are reports of a homogeneous, hyperintense late enhancement due to gadolinium accumulation in fibroelastic tissue, which is characteristically surrounded by a halo of intensity similar to that of the myocardium.^20,23,24^

## Cardiac computed tomography

Due to technological advancements, multi-slice cardiac computed tomography (CT) now acquires large volumes in a single breath-hold. This feature allows for the avoidance of motion artefacts, submillimetre thicknesses, and the ability to reconstruct 3D images.^25,26^

PFE are difficult to detect using tomography due to their size and hypermobility. Once visualized, they appear as small, hypodense masses with tentacle-like borders due to the presence of a pedicle.^27–29^ Motility is assessed using retrospective gated acquisitions of a complete heartbeat, which is important for evaluating the risk of embolization. A major advantage of this technique is its ability to provide a complementary assessment of coronary anatomy. Finally, anatomical information of both the mass and the coronary tree is important for preoperative planning.^28,30^

## Surgical intervention

The recommended treatment for left-sided PFE causing embolic symptoms is surgical resection.^1,15^ For incidentally diagnosed tumours smaller than 10 mm, non-mobile, or located on the right side, the management remains uncertain.^15^ A single-centre retrospective study reported a reduction in the rate of neurological events with surgical excision of left-sided PFEs, regardless of size, mobility, or location.^31^ The most studied surgical technique is valve-preserving resection via median sternotomy or mini-thoracotomy, which has very low and similar perioperative mortality rates, and complications are very rare. The recurrence rate is less than 5% in most series, although Mazur et al. found in one series of cases a recurrence of approximately 15% at 10 years, which seems to depend on the number of tumours or incomplete resections.^32,33^

PFE is a rare primary cardiac tumour with benign histology, but it can potentially lead to serious complications, including stroke. Multimodal imaging is crucial for diagnosis, integrating anatomical, histological, and functional characteristics, and for guiding decisions regarding surgical resection.

## Supplementary Material

qyag133_Supplementary_Data

## Data Availability

The data underlying this article are available in the article.

## Lead Author Biography

Angela Cachicatari is a cardiologist at Guillermo Almenara Irigoyen Hospital, Lima-Perú. She specializes in cardiac CT and MRI, and nuclear cardiology. She trained at the Ignacio Chavez Institute of Cardiology in Mexico. Her work focuses on imaging support in interventional cardiology.
